# Rhabdomyolysis in COVID-19 Patients: A Retrospective Observational Study

**DOI:** 10.7759/cureus.12552

**Published:** 2021-01-07

**Authors:** Magued W Haroun, Vladyslav Dieiev, John Kang, Mali Barbi, Seyed Farzad Marashi Nia, Mohamed Gabr, Gerardo Eman, Grace Kajita, Kristin Swedish

**Affiliations:** 1 Internal Medicine, Montefiore Medical Center, Wakefield Campus, Bronx, USA

**Keywords:** covid-19, acute kidney injury, rhabdomyolysis, hemodialysis, muscle breakdown

## Abstract

Background

The coronavirus disease 2019 (COVID-19) pandemic has caused significant morbidity and mortality worldwide. Knowledge about the pathophysiology of the disease and its effect on multiple systems is growing. Kidney injury has been a topic of focus, and rhabdomyolysis is suspected to be one of the contributing mechanisms. However, information on rhabdomyolysis in patients affected by COVID-19 is limited. We aim to describe the incidence, clinical characteristics, and outcomes of patients hospitalized with COVID-19 who developed rhabdomyolysis.

Materials and methods

A retrospective observational cohort consisted of patients who were admitted and had an outcome between March 16 to May 27, 2020, inclusive of those dates at a single center in the Bronx, New York City. All consecutive inpatients with lab-confirmed COVID-19 were identified. Patients with peak total creatine kinase (CK) over 1,000 U/L were reviewed; 140 patients were included in the study. The main outcomes during hospitalization were new-onset renal replacement therapy and in-hospital mortality.

Results

The median age was 68 years (range: 21-93); 64% were males. The most common comorbidities were hypertension (73%), diabetes mellitus (47%), and chronic kidney disease (24%). Median CK on admission was 1,323 U/L (interquartile range [IQR]: 775 - 2,848). Median CK on discharge among survivors was 852 (IQR: 170 - 1,788). Median creatinine on admission was 1.78 mg/dL (IQR: 1.23 - 3.06). During hospitalization, 49 patients (35%) received invasive mechanical ventilation, 24 patients (17.1%) were treated with renal replacement therapy (RRT), and 66 (47.1%) died.

Conclusions

Rhabdomyolysis was a common finding among hospitalized patients with COVID-19 in our hospital in the Bronx. The incidence of new-onset renal replacement therapy and in-hospital mortality is higher in patients who develop rhabdomyolysis. McMahon score, rather than isolated creatine kinase levels, was a statistically significant predictor of new-onset RRT. Clinicians should maintain a high level of suspicion for rhabdomyolysis in COVID-19 patients throughout their admission and use validated scores like McMahon score to devise their treatment plan accordingly.

## Introduction

Rhabdomyolysis is a syndrome characterized by skeletal muscle breakdown with the resultant release of intracellular contents into the bloodstream leading to systemic complications, most commonly acute kidney injury (AKI). Creatine kinase (CK) is the most sensitive indicator of muscle damage and has been used for laboratory confirmation of rhabdomyolysis [1]. It has been widely accepted that CK levels more than five times the upper limit of normal (≥1,000 U/L) are diagnostic of rhabdomyolysis.

There are multiple causes of rhabdomyolysis. Viral infections causing rhabdomyolysis are more common in the pediatric population but have been reported frequently also in adults. Many viral, bacterial, fungal, and protozoal infections have been associated with rhabdomyolysis, of which influenza A is the most commonly implicated [2]. More recently, rhabdomyolysis has been reported as a complication of the severe acute respiratory syndrome (SARS) caused by SARS-CoV [3]. The exact mechanism of muscle damage in viral infections has not been established.

The world is currently facing a global health crisis caused by severe acute respiratory syndrome coronavirus 2 (SARS-CoV-2). Understanding of the full spectrum of coronavirus disease 2019 (COVID-19), the disease caused by the new virus, is evolving. Since the beginning of the pandemic, it was noted that CK elevation above the upper limit of normal (>185 U/L) correlated with severe disease and increased mortality [4]. In one study, serum CK decline correlated with viral mRNA elimination [5]. However, rhabdomyolysis in COVID-19 patients has not been commonly reported - a few retrospective cohorts reported peak CK over 1,000 U/L and to date, few case reports have described patients who developed severe rhabdomyolysis (peak CK of approximately 10,000 U/L) during the course of illness [6-10].

Kidney involvement is common in COVID-19. The spectrum ranges from mild proteinuria to progressive AKI requiring renal replacement therapy (RRT) [11]. The pathophysiology of AKI is likely multifactorial and is under active investigation. Rhabdomyolysis is suspected to be one of the contributors to AKI in COVID-19 patients. In a post-mortem renal histopathological analysis of COVID-19 patients in China, pigmented casts were found with high CK levels in some cases, possibly representing rhabdomyolysis [12].

A better understanding of the incidence of rhabdomyolysis in COVID-19 patients and its clinical implications is important to fully appreciate the spectrum of damage caused by the virus and guide decision making regarding fluid management and prognostication. Our study aims to shed light on the burden of rhabdomyolysis in COVID-19 patients and to describe the demographic and clinical characteristics, laboratory findings, and outcomes among patients who presented with or developed this condition during their admission in a hospital at one of the hardest hit boroughs of New York City.

## Materials and methods

This is a retrospective observational cohort study. We obtained data from a teaching hospital (Montefiore Medical Center [MMC] - Wakefield campus) in the Bronx borough of New York City on hospitalized patients and had an outcome between March 16 and May 27, 2020. Patients were found by applying search criteria to the hospital’s electronic medical records (EMR) registry. We then obtained information by manual chart review on patient demographics (age, gender, body-mass index [BMI], and race/ethnicity), presenting symptoms, vital status at hospital discharge, electronic discharge summaries, and inpatient laboratory values. Approval for this study was granted by the institutional review board at Albert Einstein College of Medicine; the need for informed consent was waived because of the retrospective nature of the study.

Inclusion criteria were age older than 18 years, lab-confirmed COVID-19 (positive result for SARS-CoV-2 in reverse transcriptase-polymerase chain reaction assay of nasal or nasopharyngeal swabs), and peak total CK level more than 1,000 U/L during hospitalization. We excluded patients with end-stage renal disease (ESRD) and those who had evidence of a secondary cause of rhabdomyolysis, including seizures, acute coronary syndrome or crush injuries prior to admission. Primary outcomes were in-hospital mortality and new-onset RRT. The only RRT modality offered in our campus was intermittent hemodialysis.

AKI was defined by Kidney Disease Improving Global Outcomes (KDIGO) criteria [13]. Baseline creatinine was determined by reference to preadmission creatinine value within six months of admission or, if not available, the lowest creatinine measured during hospitalization. McMahon score was calculated for every patient on admission. It includes eight variables: age, gender, etiology, and initial levels of CK, creatinine, calcium, phosphate, and serum bicarbonate. McMahon score has been validated as an instrument for predicting mortality and AKI. A score greater than six is 86% sensitive and 68% specific for identifying patients who will require RRT [14-15].

Eight-hundred twenty-five patients were admitted to MMC Wakefield hospital during the study period with laboratory-confirmed COVID-19. One-hundred sixty-five patients (17%) met the criteria for rhabdomyolysis diagnosis (CK >1,000 U/L) during their admission. Twenty-five patients were excluded based on exclusion criteria mentioned prior; 140 patients were included in the study.

This research was done without patient involvement. Patients were not invited to comment on the study design and were not consulted to develop patient-relevant outcomes or interpret the results. Patients were not invited to contribute to the writing or editing of this document for readability or accuracy.

Descriptive statistics were used to summarize the data; results are reported as medians and interquartile ranges (IQR) or means and standard deviations, as appropriate. Categorical variables were summarized as counts and percentages. Univariate logistic regression was used to assess the effect of demographics, comorbidities, laboratory data, and invasive mechanical ventilation on mortality and new-onset RRT; multivariate models were created including all variables with univariate p-values <0.2. All statistical analyses were performed using SPSS statistics software (IBM Inc., Armonk, USA). No imputation was made for missing data.

## Results

Demographic and clinical characteristics

Demographics and clinical characteristics of our patients are shown in Table 1. The median age was 68 years (range: 21-93); 64% were men. The most common presenting symptoms were shortness of breath (62%), fever (50%), and cough (49%). The most common chronic medical conditions in this population were hypertension (72%), diabetes mellitus (47%), and chronic kidney disease (24%). Forty-one patients (29%) were taking statins prior to admission. Forty-nine patients (35%) received treatment in the intensive care unit for at least 24 hours.

**Table 1:** Clinical characteristics of the patients at baseline

Characteristic	Patients (N=131)
Median age (range) – yr.	68 (21-93)
Gender – no. (%)	
Male	90 (64.3)
Female	50 (35.7)
BMI – median (IQR)	29.5 (25.2-35.1)
Race and ethnicity – no. (%)	
African American	85 (60.7)
Hispanic	29 (20.7)
White	7 (5)
Asian	1 (0.7)
Missing or other	18 (12.9)
Coexisting disorder – no. (%)	
Hypertension	101 (72.1)
Diabetes mellitus	65 (46.4)
Chronic kidney disease	34 (24.3)
Dementia	22 (15.7)
Asthma	17 (12.1)
Hemorrhagic or ischemic stroke	13 (9.3)
Chronic obstructive pulmonary disease	12 (8.6)
Atrial fibrillation	7 (5)
Coronary artery disease	7 (5)
Seizure disorder	5 (3.6)
Statin use prior to admission – no. (%)	41 (29.3)
Respiratory symptoms – no. (%)	
Shortness of breath	87 (62.1)
Cough	68 (48.6)
Systemic symptoms – no. (%)	
Fever	70 (50)
Altered mental status	26 (18.6)
Diarrhea	16 (11.4)
Weakness	18 (12.9)
Myalgias	12 (8.6)
Chest pain	10 (7.1)

Laboratory results

Laboratory data during hospitalization are summarized in Table 2. The total CK lab assay at our institution has a maximum limit of 80,000 U/L. Results greater than 80,000 U/L are reported as (>80,000 U/L). Median CK on admission was 1,323 U/L (IQR: 775 - 2,848). Median CK on discharge among survivors was 852 (IQR: 170 - 1,788). Median creatinine on admission was 1.78 mg/dL (IQR: 1.23 - 3.06).

**Table 2:** Laboratory data during hospitalization IQR - interquartile range; FEU - fibrinogen equivalent units

Laboratory Data	Patients (N=140)	Reference range
Creatine kinase – U/L		
Admission, median (IQR)	1,323 (775 - 2,848)	<200 U/L
Peak, median (IQR)	2,209 (1,283 - 4,161)	<200 U/L
Creatinine – mg/dL		
Admission, median (IQR)	1.78 (1.23 - 3.06)	<1.20 mg/dL
Peak, median (IQR)	3.02 (1.60 - 6.48)	<1.20 mg/dL
C-Reactive Protein – mg/dL		
Peak, median (IQR)	21.2 (10.9 - 33.1)	<0.8 mg/dL
D-Dimer – ug/mL FEU		
Peak, median (IQR)	6.88 (2.29 - 20)	0.00 - 0.05 ug/mL FEU
McMahon’s score on admission		
<6, no. (%)	31 (22.1)	
≥6, no. (%)	109 (77.9)	

Outcomes

Out of the 140 patients who were included in this study, 74 (52.9%) were discharged, and 66 (47.1%) died in the hospital. One-hundred-and-twelve patients (80%) developed AKI, twenty-four (17.1%) of which required RRT. Five patients were planned for RRT but expired prior to RRT initiation or family refused RRT. All patients planned for RRT were included in the final analysis. Results of our univariate and multivariable logistic regression model for mortality and new-onset RRT are summarized in Tables 3 and 4, respectively.

**Table 3:** Predictors of mortality OR - odds ratio; CI - confidence interval; BMI - body mass index; AKI - acute kidney injury; RRT - renal replacement therapy

	Univariate OR (95% CI)	p-value	Multivariate OR (95% CI)	p-value
Demographics				
Age	1.05 (1.02 - 1.08)	0.000	1.06 (1.01 - 1.12)	0.027
Male gender	1.35 (0.68 - 2.71)	0.391		
BMI	0.99 (0.96 - 1.04)	0.968		
Co-morbidities				
Hypertension	2.21 (1.02 - 4.78)	0.044		
Diabetes mellitus	1.82 (0.93 - 3.57)	0.082		
Chronic kidney disease	1.41 (0.64 - 3.07)	0.393		
Dementia	2.82 (1.07 - 7.41)	0.036	7.72 (1.34 - 44.46)	0.022
Laboratory measures				
Creatine kinase				
Admission	1.00 (1.00 - 1.00)	0.109		
Peak	1.00 (1.00 - 1.00)	0.594		
Time to peak	1.07 (0.96 - 1.17)	0.194		
Creatinine				
Admission	1.04 (0.92 - 1.18)	0.520		
Peak	1.13 (1.03 - 1.24)	0.005		
C-Reactive protein, peak	1.06 (1.03 - 1.20)	0.000	1.06 (1.01 - 1.11)	0.011
D-Dimer, peak	1.12 (1.05 - 1.18)	0.000		
McMahon’s score ≥6	2.68 (1.13 - 6.34)	0.025		
Clinical measures				
AKI	2.69 (1.09 - 6.60)	0.031		
New-onset RRT	2.12 (0.86 - 5.25)	0.102	3.77 (0.83 - 17.13)	0.085
Invasive mechanical ventilation	8.69 (3.80 - 19.84)	0.000	16.34 (4.43 - 60.23)	0.000

**Table 4:** Predictors of new-onset RRT* *Including patients who were planned for RRT but expired prior to the actual initiation of RRT. **Creatinine values were not included in this multivariate model to avoid confounding. OR - odds ratio; CI - confidence interval; BMI - body mass index; RRT - renal replacement therapy

	Univariate OR (95% CI)	p-value	Multivariate OR (95% CI)	p-value
Demographics				
Age	0.98 (0.95 - 1.01)	0.161	0.893 (0.838 - 0.951)	0.000
Male gender	0.63 (0.25 - 1.54)	0.308		
BMI	1.04 (0.99 - 1.09)	0.099		
Co-morbidities				
Hypertension	2.88 (0.93 - 8.90)	0.066	64.84 (2.72 - 1548.14)	0.010
Diabetes mellitus	6.20 (2.34 - 16.49)	0.000	5.32 (1.07 - 26.51)	0.041
Chronic kidney disease	1.74 (0.69 - 4.37)	0.242		
Dementia	0.34 (0.07 - 1.53)	0.160		
Laboratory measures				
Creatine kinase				
Admission	1.00 (0.99 - 1.00)	0.100	0.99 (0.99 - 1.00)	0.058
Peak	1.00 (1.00 - 1.00)	0.620		
Time to peak	1.17 (1.05 - 1.29)	0.003		
Creatinine**				
Admission	1.37 (1.16 - 1.62)	0.000		
Peak	2.40 (1.70 - 3.38)	0.000		
C-Reactive protein, peak	1.04 (1.01 - 1.07)	0.007		
D-Dimer, peak	1.05 (0.99 - 1.11)	0.100		
Clinical measures				
McMahon’s score ≥6	10.37 (1.35 - 79.61)	0.024	100.21 (4.53 - 2215.1)	0.004
Invasive mechanical ventilation	5.37 (2.24 - 12.89)	0.000		

Outcomes as compared to MMC Wakefield COVID-19 admissions who had peak CK less than 1,000 U/L during the same period are summarized in Table 5. The incidence of rhabdomyolysis among all COVID-19 patients admitted to our hospital during the study period was 16.9%. The in-hospital mortality rate in COVID-19 patients who developed rhabdomyolysis was significantly higher than mortality among COVID-19 patients who did not develop rhabdomyolysis (47.1% vs. 26.4%). The incidence of new-onset RRT among COVID-19 patients who developed rhabdomyolysis was over triple the incidence in COVID-19 patients who did not develop rhabdomyolysis (17.1 vs. 2.9%).

**Table 5:** Outcomes as compared to all COVID-19 admissions during the same time period CK - creatine kinase; RRT - renal replacement therapy; COVID-19 - coronavirus disease 2019

	Patients admitted with COVID-19 and peak CK <1000 U/L	Study group: patients admitted with COVID-19 and peak CK >1000	Total
N	685	140	825
New-onset RRT – no. (%)	20 (2.9)	24 (17.1)	44 (5.3)
In-hospital mortality – no. (%)	181 (26.4)	66 (47.1)	247 (29.9)

Most patients (n=132, 94.3%) in our cohort met the definition of rhabdomyolysis (CK greater than 1,000 U/L) within the first week of admission, while seven patients developed rhabdomyolysis during their second week of admission. Notably, all seven patients were intubated. In total, 49 patients (35%) were intubated and mechanically ventilated. The mortality rate among intubated patients was 79.2%.

## Discussion

This study represents the first observational study providing a snapshot of the burden of rhabdomyolysis among COVID-19 patients admitted to one hospital in one of the most affected boroughs at the pandemic epicenter.

Patients who developed rhabdomyolysis had higher rates of mortality and new-onset RRT as compared to COVID-19 patients with peak CK less than 1,000 U/L. However, based on our logistic regression models, CK levels on admission and peak CK levels were not found to be statistically significant predictors of either new-onset RRT or mortality in our study group. McMahon’s score on admission had the strongest correlation with new-onset RRT, highlighting its prognostic value in COVID-19 patients with rhabdomyolysis as well as its role in guiding the initiation of renal protective therapies. Other statistically significant predictors of new-onset RRT were young age, diabetes mellitus, hypertension, and admission creatinine. We suspect a young age was a significant predictor because younger patients were more likely to receive more aggressive treatment modalities, including RRT.

In our multivariate model for mortality, age, dementia, peak C-reactive protein (CRP) levels, and mechanical ventilation were found to be statistically significant predictors of mortality. These findings support recent reports correlating age, CRP levels, and dementia with mortality [16-17]. CRP levels and mechanical ventilation likely reflect critical illness leading to higher mortality.

The mechanism of muscle damage in viral infections, particularly COVID-19, is not fully understood. Proposed hypotheses include direct and indirect mechanisms. The first theory is via direct viral muscle invasion. Angiotensin-converting enzyme 2 (ACE2), which was identified as the functional receptor for SARS-CoV and SARS-CoV-2, is present in skeletal muscles [18-19]. SARS-CoV was not detected in skeletal muscle by postmortem examination, and to this date, it is unclear if SARS-CoV-2 infects muscles directly [20]. The second theory postulates that skeletal muscle damage could be caused by the host “cytokine storm”-like immune response [2].

Serum CK usually begins to rise approximately two to 12 hours after onset of muscle injury, peaks within 24 to 72 hours, and then declines gradually over 7-10 days unless there is continuing muscle injury or development of compartment syndrome [1]. While CK is widely used for laboratory diagnosis of rhabdomyolysis, kidney injury is believed to be caused by myoglobin, which peaks and drops much faster than CK. In two published clinical vignettes, COVID-19 patients who developed rhabdomyolysis were reported on admission in one case and as a late complication (on day nine of hospitalization) in another [8-9]. Most patients (94%) in our cohort had peak CK on the presentation or within one week of admission, suggesting the onset of muscle injury likely took place around the time they were admitted to the hospital. Only 5% of patients in our cohort developed late rhabdomyolysis during admission.

Our study had several limitations. First, all data were retrospectively obtained from electronic medical records, so some demographic and clinical data were missing. Second, we were unable to retrieve data pertaining to treatments given to the patients, especially the rate and timing of intravenous hydration. Obtaining accurate fluids data retrospectively would be challenging. Third, the only RRT modality offered in our campus was intermittent hemodialysis, limiting access for hemodynamically unstable patients. Fourth, resource allocation during a time of crisis like this pandemic might have resulted in a lack of hemodialysis treatment or shortened hemodialysis time, which could have impacted mortality rates. Fifth, for some predictors of outcomes in our models, the confidence interval was very wide, likely secondary to small sample size. Larger studies are warranted.

## Conclusions

In conclusion, rhabdomyolysis is an important aspect of the multifaceted nature of COVID-19 and is likely underreported. CK levels in isolation might not correlate with outcomes, so clinicians are encouraged to use validated scores like McMahon score to better predict outcomes and guide treatment decisions for patients who develop rhabdomyolysis, most importantly their fluid management, which can be challenging in patients with impending respiratory failure and should be decided on a case-by-case basis.
